# Predictors of delayed vaccination in infants born in Tuscany, Italy: an area based cohort study

**DOI:** 10.1093/eurpub/ckac129.392

**Published:** 2022-10-25

**Authors:** V Lastrucci, M Puglia, M Pacifici, F Rusconi, P Buscemi, G Alderotti, M Sica, G Belli, E Berti, F Voller

**Affiliations:** 1 Epidemiology Unit, Meyer Children's Hospital, Firenze, Italy; 2 Epidemiologic Observatory, Regional Health Agency of Tuscany, Firenze, Italy; 3 Maternal and Child Department, Local Health Unit Toscana Nord Ovest, Pisa, Italy; 4 Department of Health Science, University of Florence, Firenze, Italy; 5 Neonatal Intensive Care Unit, Local Health Unit Toscana Centro, Firenze, Italy; 6 Neonatal Intensive Care Unit, Meyer Children's Hospital, Firenze, Italy

## Abstract

**Background:**

Timely vaccination is essential to protect infants from vaccine-preventable diseases. The aim of the study was to evaluate the determinants of vaccination timeliness for hexavalent (HEXA) and measles-mumps-rubella (MMR) vaccines.

**Methods:**

The study is part of the PREHMO project funded by Tuscany Region, Italy. Data on the 2017 and 2018 full birth cohorts of Tuscany (N = 41,493) were retrieved from the Birth Registry and linked to those of the Vaccine Registry up to 24 months after birth. Sociodemographic and at birth characteristics of mothers and infants were retrieved. The primary outcome was the timeliness of HEXA 1st and 3rd doses, and MMR 1st dose. Timeliness was defined as the administration of the dose a day after the period recommended by the vaccination schedule. Multiple logistic regression models were performed.

**Results:**

For all the vaccines considered, a significantly increased risk of delayed vaccination was observed in preterm infants and in infants born in hospital of second level of newborn care, while infants conceived by assisted reproductive technologies and first-born infants showed a significantly decreased risk for delayed vaccination. Multiple births, small for gestational age status, maternal unemployment, and rural residence were significantly associated with an increased risk of delayed HEXA-1 vaccination (OR 1.31, 95%CI 1.13-1.51; OR 1.12, 95%CI 1.03-1.22; OR 1.06, 95%CI 1.01-1.13; and OR 1.1, 95%CI 1.03-1.16). As for MMR vaccination, a low maternal education was significantly associated with high risk of delay (OR 1.12, 95%CI 1.06-1.18), while rural residence, maternal foreign nationality and female sex were significantly associated with a decreased risk of delay (OR 0.91, 95%CI 0.87-0.96; OR 0.82, 95%CI 0.78-0.87; and OR 0.95, 95%CI 0.91-0.99).

**Conclusions:**

Several common and vaccine-specific predictors of vaccination timeliness were identified. Strategies to improve a timely vaccination should take into account these predictors.

**Key messages:**

• Several maternal and infants factors may influence vaccination timeliness of routine immunization in childhood.

• Tailored vaccination strategies are needed to improve vaccination timeliness in infants at high-risk of delayed vaccination.

